# Bone metabolism in complex regional pain syndrome

**DOI:** 10.1097/PR9.0000000000001217

**Published:** 2024-11-20

**Authors:** Michael A. Harnik, Annemarie Sodmann, Beate Hartmannsberger, Gudrun Kindl, Juliane Becker, Ann-Kristin Reinhold, Eva Herrmann, Andreas K. Buck, Ulrich Dischinger, Frank Birklein, Alexander Brack, Abdelrahman Sawalma, Heike L. Rittner

**Affiliations:** a Department of Anaesthesiology, Intensive Care, Emergency and Pain Medicine, Centre for Interdisciplinary Pain Medicine, University Hospital Würzburg, Würzburg, Germany; b Department of Anaesthesiology and Pain Medicine, Inselspital, Bern University Hospital, University of Bern, Bern, Switzerland; c Departments of Neurology and; d Nuclear Medicine, University Hospital Würzburg, Würzburg, Germany; e Division of Endocrinology and Diabetes, Department of Internal Medicine, University Hospital Würzburg, Würzburg, Germany; f Department of Neurology, University Medical Centre of the Johannes Gutenberg University Mainz, Mainz, Germany

**Keywords:** Complex regional pain syndrome, Bone markers, Three-phase bone scintigraphy, Biomarkers

## Abstract

**Introduction:**

Patients with complex regional pain syndrome (CRPS) often show disturbed bone metabolism, assessed using three-phase bone scintigraphy (TPBS). However, current methods lack automation and standardisation. Bone serum markers have been proposed as biomarkers, but their utility is unclear.

**Objectives:**

This study aimed to evaluate bone metabolism in CRPS using TPBS and bone serum markers.

**Methods:**

A deep learning model for automated segmentation quantified tracer enhancement in TPBS images. Serum markers analysed included alkaline phosphatase (AP), 25-OH vitamin D, osteoprotegerin, procollagen type I N-terminal propeptide (PINP), and β-C-terminal telopeptide, compared to 48 healthy controls (HC). The study included 114 patients with CRPS, 41 of whom underwent TPBS.

**Results:**

Of the 41 patients with CRPS with TPBS, 39 (95.1%) displayed radiotracer enhancement in the bone phase across CRPS subtypes. Serum markers of 114 patients did not significantly differ between patients and HC, nor did they correlate with TPBS enhancement, except in warm CRPS. In these patients, TPBS accumulation in the metacarpophalangeal region correlated with PINP (Spearman ρ = 0.63, *P* = 0.038), and AP levels were elevated at 78 U/L (interquartile range 64–88) compared to cold CRPS at 66 U/L (51–77; *P* = 0.003) and HC at 60 U/L (53–69; *P* < 0.001).

**Conclusion:**

Automated TPBS quantification revealed widespread bone metabolism alterations, common in CRPS and detectable beyond qualitative assessment. Although most serum markers remained unchanged, patients with warm CRPS exhibited unique features, suggesting distinct pathophysiological profiles. Integrating novel image analysis with other biomarkers may enhance diagnostic precision and patient stratification for targeted therapies.

## Abstract 2

Supplemental Digital Content is Available in the Text.

Automated quantification of three-phase bone scintigraphy of 41 patients with complex regional pain syndrome demonstrated frequently altered bone metabolism across subtypes. Blood bone biomarkers were less important.

## 1. Introduction

Complex regional pain syndrome (CRPS), a rare chronic pain disorder that typically occurs after trauma to the extremities, is diagnosed using the clinical Budapest criteria.^13^ However, confirming the diagnosis remains challenging owing to the lack of objective measures and variable pathophysiological mechanisms across subtypes and stages. These complexities may contribute to the variable efficacy of pharmacological trials such as bisphosphonates in large populations. Therefore, identifying precise CRPS biomarkers is crucial for patient stratification and development of targeted therapies.

Distinctions within CRPS include (1) warm and cold subtypes, (2) CRPS I and II, (3) central and peripheral CRPS, and (4) acute and chronic CRPS.^9,18^ Recently, bone-related biomarkers have been proposed to identify patients with increased bone metabolism.^21^ Traditional examinations such as three-phase bone scintigraphy (TPBS) capture local bone turnover through radiotracer accumulation; however, their qualitative assessment lacks the sensitivity needed for a definitive diagnosis.^47^ Currently, no standardised automated method exists for quantifying TPBS in patients with CRPS.

Alternatively, bone serum markers have been suggested as potential biomarkers of bone metabolism.^21^ For instance, osteoprotegerin (OPG) was significantly elevated in acute CRPS and moderately correlated with TPBS in a smaller study, indicating increased osteoblast activity during the early disease stage.^23^ Furthermore, alkaline phosphatase (AP) levels were elevated in acute but not chronic CRPS phases in 2 small cohorts.^30,39^ Other serum markers (calcium, phosphate, 25-OH vitamin D, and parathyroid hormone) remained unchanged.^30^ In addition, collagen type I byproducts have gained interest, as procollagen type I N-terminal propeptide (PINP) and β-C-terminal telopeptide (β-CTx) can be used to assess bone formation and degradation. However, their role in CRPS remains unexplored.^21^

This study aimed to quantify the prevalence of disturbed bone metabolism in CRPS using an advanced automated TPBS assessment and to validate various bone serum markers in a comprehensive study population. In addition, we evaluated whether these markers could supplement or replace TPBS. Finally, we aimed to identify a CRPS subgroup with disturbed bone metabolism to enhance the diagnostic precision and stratification for targeted treatment approaches.

## 2. Methods

### 2.1. Participants

This single-centre cross-sectional study was part of the multicentre clinical research unit ResolvePAIN (KFO5001) funded by the German Research Foundation (Deutsche Forschungsgemeinschaft [DFG]). A total of 120 patients with CRPS were prospectively enrolled, after approval from the ethics committee responsible for the region (German Trial Registration DRKS00016790). All the patients provided written informed consent in accordance with the Declaration of Helsinki. Inclusion criteria were patients of both sexes, aged 18 to 85 years, and diagnosed with CRPS according to the clinical Budapest criteria.^13^ Exclusion criteria were active autoimmune diseases (eg, systemic lupus erythematosus) or severe neurological diseases (eg, amyotrophic lateral sclerosis), recent infection, history of cancer or bone disease, and surgery within the previous 4 weeks. Kidney and liver functions were assessed, and patients with pathological results were excluded. No patients received TPBS before their baseline visit, preventing referral bias. Clinical phenotyping was supplemented with TPBS during the study, and serum markers AP, 25-OH vitamin D, OPG, PINP, and β-CTx were determined. Patients could consent to or reject any part of the study. Serum values from 48 healthy controls (HC), initially recruited for another study via newspaper advertising and within the clinic, were used for comparison.

### 2.2. Clinical examination and questionnaires

Complex regional pain syndrome severity was assessed using the 16-item CRPS Severity Score (CSS).^14^ Pain intensity and functional interference were measured using the Graded Chronic Pain Scale (GCPS) with a numeric rating scale (0 = no pain/no interference; 10 = worst pain imaginable/unable to carry on any activities).^22^ Patients underwent nerve conduction studies to distinguish CRPS type II from type I. Furthermore, patients were stratified into “warm” and “cold” subtypes, confirmed by an infrared temperature difference of ≥1°C on the dorsum of the hand or foot after at least 30 minutes of acclimatization. Differences of <1°C were labelled as “intermediate”. Quantitative sensory testing (QST) was performed according to the standards of the German Research Network on Neuropathic Pain,^34^ focusing on pressure pain thresholds,^26^ using a handheld pressure algometer over the thenar muscles (force dial FDK/FDN series mechanical force gauge; Wagner Ins, Greenwich, CT). Measurements, recorded in kPa and averaged over 3 repetitions, were z-transformed based on age, sex, and body area (ie, hands or feet) using published reference data.^25^ Medical records were reviewed for documentation of peripheral injuries and medication intake (antidepressants, gabapentinoids, bisphosphonates, and glucocorticoids).

### 2.3. Three-phase bone scintigraphy analysis

To perform an objective TPBS analysis, a new tool using a deep learning approach was developed. The contralateral, unaffected side served as a control for the ipsilateral side, as previous research demonstrated no significant difference in radiotracer accumulation between the unaffected limbs in patients with CRPS and controls.^23^ Three-phase bone scintigraphy images were extracted in the TIFF format from medical records, together with their qualitative analysis by a board-certified nuclear medicine physician. Images of all 3 phases (phase 1, flow phase; phase 2, blood pool phase; phase 3, delayed/bone phase) were cut, resized to 400 × 400 pixels, and saved in the TIFF format using ImageJ2.^36^ Regions of interest (ROI) for the affected and unaffected sides were annotated using QuPath.^3^ Owing to difficulties in separating joints in the feet, the entire foot was annotated for phases 1 to 3 of the lower extremities. For the upper extremities, the whole hand was annotated for phases 1 to 2, and the carpal, metacarpophalangeal (MCP), and proximal interphalangeal (PIP) subregions for phase 3.

After splitting the data into training (70%), validation (20%), and test data (10%), 3 neural network models with U-net architecture were trained. An iterative active learning approach was used along with an uncertainty measure for out-of-distribution detection.^12^ Incorrect predictions were relabelled and retrained until sufficient model performance was achieved. This was measured using the Dice coefficient to validate the pairwise overlap between manual annotation and automated segmentation, with scores ranging from 0 (no congruence) to 1 (100% congruence).^7,52^ The source code is available on GitHub.^41^

Pixel intensities (0–255) were calculated for all ROIs and inverted for better interpretability. Ratios of ipsilateral to contralateral sides were computed for all phases and regions, with values > 1 indicating a greater accumulation on the affected side.

### 2.4. Bone serum markers

Serum analyses of bone status were conducted by measuring AP, 25-OH vitamin D, OPG, and the 2 collagen I turnover byproducts, PINP and β-C-terminal telopeptide (β-CTx). Alkaline phosphatase correlates with the number of circulating osteoblasts and thus indirectly with bone formation,^16^ whereas high AP is linked to lower bone mineral density.^40^ Vitamin D deficiency is associated with reduced bone density and pain-related changes such as central sensitisation.^2^ Osteoprotegerin prevents osteoclast activation and was elevated in patients with CRPS compared to fracture patients and controls in a pilot study.^6,19,23,42^ Procollagen type I N-terminal propeptide is cleaved off procollagen I by osteoblasts during bone formation,^43^ whereas β-CTx are peptide fragments from fibrillary collagens that are degraded by osteoclasts during bone resorption. Both PINP and β-CTx can be used to detect bone metabolism changes after 3 months and are sensitive to antiresorptive therapy.^16,45^

Blood samples were collected in the morning after overnight fasting (Sarstedt S-monovette EDTA, 9 mL; Sarstedt, Nürnbrecht, Germany). Alkaline phosphatase was measured using routine tests. Further samples were centrifuged at room temperature at 1300 rpm for 10 minutes, and the resulting supernatant serum was stored in 2 mL Eppendorf caps at −80°C. As nonthawed serum was not available for each participant, the group sizes in serum analyses differed slightly for AP (n = 114), PINP and β-CTx (n = 113), and OPG (n = 108). Serum values were plotted against storage time to check for bias caused by longer freezing times. Osteoprotegerin levels were determined using an enzyme-linked immunospecific assay (Immundiagnostik, Bensheim).^23^ Triple-sample optical densities were analysed using the assayfit pro Excel Plugin Version 1.4^44^ and Python's curve_fit function. The results from both methods were identical up to the third decimal place, and assayfit values were used. Blank reference values were subtracted, and measures were converted to pg/mL (0.05 pmol/L = 1 pg/mL). Commercial chemiluminescence assays were used to measure 25 OH-vitamin D, β-CTx, and PINP (IS-2500N, IS-3000, and IS-4000, IDS, East Boldon, United Kingdom). All analyses were performed at the University Hospital Würzburg.

### 2.5. Statistical analysis

Categorical variables were analysed using counts and frequencies with Pearson χ^2^ test for group comparisons. Subgroup analyses were performed for warm and cold temperature types and CRPS types I and II. Continuous and ordinal data, such as values on a numeric rating scale, medians, and interquartile ranges (first–third quartiles), were calculated. Group comparisons were conducted using the Mann–Whitney *U* test or Kruskal–Wallis test, followed by post-hoc Dunn test with Bonferroni correction, as appropriate.

To account for potential confounders between HC and CRPS groups, cases were matched using R's MatchIt package, performing 1:1 nearest-neighbour matching based on age and sex using Euclidean distance. Immobilisation duration was explored as a potential confounder in an exploratory analysis.

Spearman correlations were used to assess the relationship between bone serum markers and TPBS ratios. In addition, as age and sex significantly influence bone turnover, they were controlled by including them as covariates in partial correlation and conditional correlation paradigms, respectively.

Statistical computations were performed using R^33^ and Python version 3.11.^35^ Statistical significance was set at *P* < 0.05.

## 3. Results

### 3.1. Study cohort and patient characteristics

Between August 2018 and October 2023, 114 patients and 48 HC were included in the final analysis. The CRPS cohort was predominantly female (74%), with a median age of 53 years, 5 months since the triggering event, and 6 weeks of immobilisation. Most patients displayed upper extremity affection (67%) and had CRPS type I (74%); 52% were categorised as having a warm CRPS (Table 1). Regarding the temperature subtypes, 59 (51.8%) patients exhibited warm CRPS. They were older, had CRPS type I less frequently than those with cold CRPS, and their upper extremities were more often affected (Table 2). No significant differences were observed in the time since triggering event or in the use of medications (antidepressants and gabapentinoids, bisphosphonates, or glucocorticoids) across the cold, intermediate, and warm subtypes.

**Table 1:** Baseline characteristics of patients and healthy controls.

	Patients with CRPS			HC N = 48	*P*
	Complete cohort N = 114	Without TPBS N = 73	With TPBS N = 41		
Age	53 (40, 60)	53 (41, 61)	52 (37, 55)	52 (37, 60)	0.2
Female	84 (74%)	55 (75%)	29 (71%)	26 (54%)	0.046
Trigger					0.7
Fracture	57 (50%)	35 (48%)	22 (54%)		
Minor trauma	6 (5.3%)	3 (4.1%)	3 (7.3%)		
Other	11 (9.6%)	7 (9.6%)	4 (9.8%)		
Surgery	40 (35.1%)	28 (38%)	12 (29%)		
Time since triggering event (d)	152 (114, 311)	158 (124, 413)	146 (99, 281)		0.2
Immobilisation duration (wk)	6 (3, 7)	6.0 (3, 6)	6 (2, 8)		0.7
Upper extremity	76 (67%)	47 (64%)	29 (71%)		0.5
Type					0.2
CRPS type I	84 (74%)	57 (78%)	27 (66%)		
CRPS type II	30 (26%)	16 (22%)	14 (34%)		
Temperature subtype					0.7
Cold	25 (22%)	14 (19%)	11 (27%)		
Intermediate	30 (26%)	20 (27%)	10 (24%)		
Warm	59 (52%)	39 (53%)	20 (49%)		
CSS total	11 (9, 13)	11 (9, 13)	12 (10, 13)		0.5
Symptoms	7 (6, 7)	6 (5, 7)	7 (6, 7)		0.5
Signs	5 (4, 6)	5 (4, 6)	5 (4, 6)		>0.9
GCPS maximum pain during the past 4 wk	8 (7, 9)	8 (6, 9)	8 (7, 9)		0.2
GCPS average pain during the past 4 wk	5 (3, 7)	5 (3, 6)	5 (4, 7)		0.11
GCPS impaired activities	6 (3, 7)	5 (3, 7)	7 (5, 8)		0.013

Demographic values of the participants. Patients with CRPS were divided into those without or with three-phase bone scintigraphy (TPBS). Values are medians (first, third quartile) or absolute and relative frequencies n (%). Comparisons were made between patients without or with TPBS and healthy controls and analysed using Mann–Whitney *U* test, Kruskal–Wallis test, or Pearson χ^2^ test.

CRPS, complex regional pain syndrome; CSS, CRPS severity score; GCPS, Graded Chronic Pain Scale on a numeric rating scale 0 to 10; HC, healthy controls.

**Table 2:** Bone parameters and medication in complex regional pain syndrome temperature subtypes.

	Cold N = 25	Intermediate N = 30	Warm N = 59	*P*
Age	43 (36, 53)	55 (42, 61)	53 (43, 61)	0.025
Female	20 (80%)	22 (73%)	42 (71%)	0.7
Time since triggering event (d)	237 (117, 485)	140 (112, 187)	155 (112, 366)	0.2
CRPS type I	22 (88%)	16 (53%)	46 (78%)	0.008
Upper extremity	11 (44%)	22 (73%)	43 (73%)	0.025
25-OH vitamin D µg/L	25 (19, 33)	25 (20, 29)	21 (15, 27)	0.2
OPG pg/mL	168 (140, 218)	167 (133, 232)	184 (156, 230)	0.6
PINP ng/mL	53 (36, 76)	58 (44, 64)	55 (48, 71)	0.8
β-CTx ng/mL	0.31 (0.20, 0.41)	0.25 (0.16, 0.36)	0.28 (0.20, 0.38)	0.8
Medication				
Antidepressants or gabapentinoids	20 (80%)	20 (67%)	37 (63%)	0.3
Bisphosphonates	0 (0%)	1 (3.3%)	0 (0%)	0.5
Glucocorticoids	6 (24%)	5 (17%)	22 (37%)	0.1

No differences were detected between the specific bone serum markers among CRPS temperature subtypes. Values are medians (first, third quartile) and absolute and relative frequencies n (%). Data were analysed using the Kruskal–Wallis test or Pearson χ^2^ test.

CRPS, complex regional pain syndrome; CSS, CRPS severity score; AP, alkaline phosphatase; OPG, osteoprotegerin; PINP, procollagen type I N-propeptide; β-CTx, β-C-terminal telopeptide.

### 3.2. Three-phase bone scintigraphy analysis

**Figure 1.**
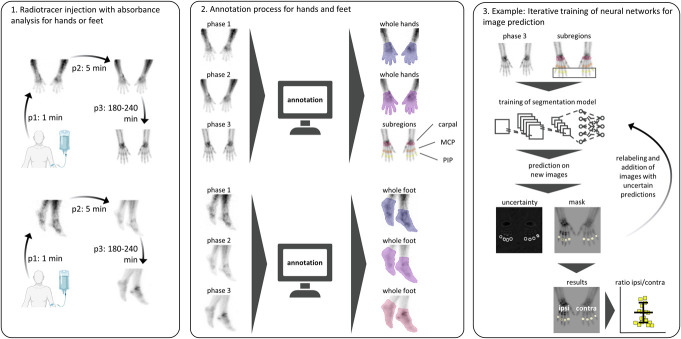
Schematic of the automated image segmentation process. (1) Illustration of image acquisition in three-phase bone scintigraphy for all 3 phases. (2) Annotation process for the hands (with their regions and subregions) and feet. (3) Example of iterative training for image segmentation and prediction of prox. interphalangeal region, and quantification of radiotracer accumulation by calculating the ratio between the mean pixel intensity values of the ipsilateral and contralateral sides. Phases 1–3, p1–p3; metacarpophalangeal and prox. interphalangeal subregions of the hands, MCP and PIP.

Three-phase bone scintigraphy was performed on 41 patients, and images and qualitative assessments were extracted from their medical records. Among them, 28 had CRPS in the upper extremities and 13 in the lower extremities. Qualitative evaluation revealed that 32 (78%) patients exhibited the characteristic scintigraphic signs of CRPS. The main reasons for rejecting the diagnosis of CRPS were as follows: *only degenerative changes* in 2 cases, *no visible accumulation* in 3 cases, and *only focal accumulation near the largest joint without peripheral distribution* in 4 cases. For quantitative analysis using automated TPBS segmentation with deep learning, training an ensemble of 3 models for both hands and feet resulted in acceptable-to-good uncertainty scores and high Dice scores, indicating excellent congruence across all phases and extremities (Table S1, http://links.lww.com/PR9/A267). A detailed schematic of the extraction, annotation, and training processes is shown in Figure 1. To facilitate future research, a graphical user interface (GUI) was developed to facilitate the use of the trained model and subsequent editing processes, allowing the analysis of single images or complete folders with TPBS images of the hands or feet (Supplementary Figure S1, http://links.lww.com/PR9/A267). The software is available free of charge for Windows and MacOS.^37,38^

**Figure 2.**
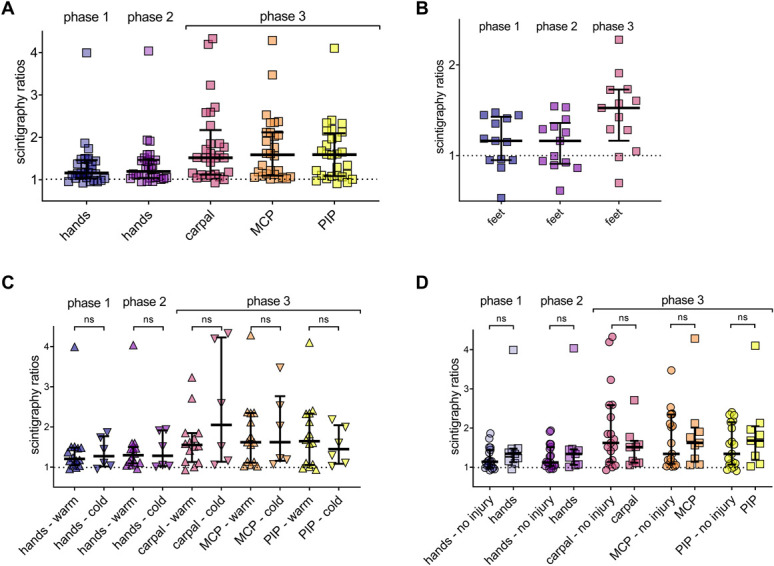
Increased bone turnover distal to the injury in almost all patients, independent of CRPS subtype and injury location. The ipsilateral/contralateral scintigraphy ratios are shown for all regions and subregions, and the dotted line indicates a ratio of 1, which is equivalent to no difference. Ratios >1 correspond to accumulation in the injured limb. Lines indicate the medians and first and third quartile. (A) Upper extremity ratios (N = 28) and (B) lower extremity ratios (N = 13). (C) No statistically significant differences were observed between the warm and cold temperature subtypes of the hands. (D) No statistically significant differences between cases with (N = 9) and without (N = 19) injury to the distal hand. Pairwise comparisons with Mann–Whitney *U* test. Carpal, metacarpophalangeal, and prox. interphalangeal subregions of the hands, carpal, MCP, and PIP.

All 3 models provided mean pixel intensity values for their respective ROI, and scintigraphy ratios >1 were observed in most cases (Figs. 2A and B). Of the 41 patients who underwent TPBS, 39 (95.1%) showed increased radiotracer uptake in at least 1 region during phase 3. The tracer accumulated in phases 1 and 2 in the hands and feet, with ratios increasing further in phase 3 in the whole foot and hand subregions, especially in the carpal, MCP, and PIP areas. The latter 2 were prominent, particularly in the MCP, where only ratios >1 were observed. These results demonstrate that quantifying TPBS using automated segmentation can reveal subtle metabolic alterations that are not apparent through visual examination alone.

Stratification by temperature subtype (warm and cold; Fig. 2C) and CRPS types I or II revealed no differences in the accumulation ratios in the hands. Among the 28 patients with upper limb CRPS, 19 (68%) had no recorded peripheral trauma to the hand, and these patients exhibited no decrease in scintigraphy ratios (Fig. 2D). Moreover, no correlations were observed between immobilisation duration and TPBS ratios of the third phase (carpal, MCP, PIP, or feet) in an exploratory analysis (all *P*-values >0.9). Deep pain, as measured by ipsilateral PPT in the QST, showed no correlation with scintigraphy ratios (see Supplementary Figure S2F, http://links.lww.com/PR9/A267 for an example of a correlation between PPT and TPBS of the carpal region). These findings suggest that radiotracer enhancement in CRPS occurs regardless of subtype, injury location, or immobilisation duration and is not evident in the upper extremities based on deep pressure pain assessment over the thenar muscle.

### 3.3. Bone serum markers

**Figure 3.**
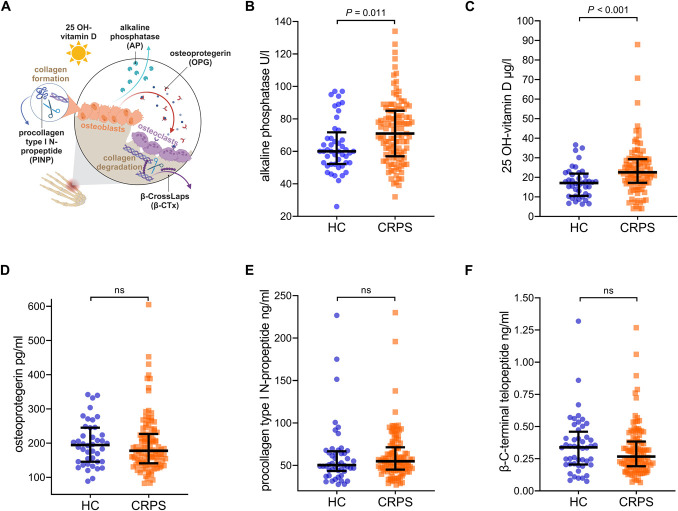
Elevated alkaline phosphatase levels in patients with CRPS, but no difference in specific bone serum markers. (A) Schematic representation of bone serum markers. (B–F) Pairwise comparisons between bone serum markers in healthy controls (HC, n = 48) and patients with CRPS (n = 114 for alkaline phosphatase). Values are the median and the first and third quartile. Pairwise comparisons with Mann–Whitney *U* test.

**Figure 4.**
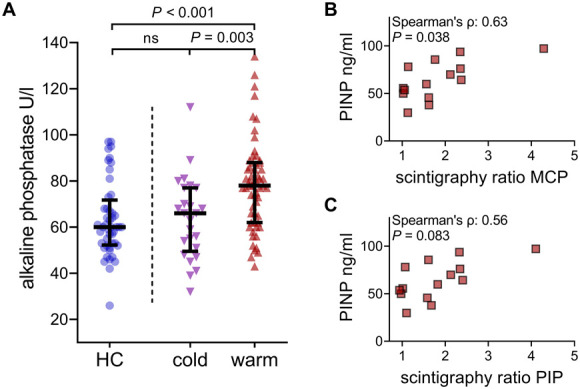
Higher AP levels in patients with warm CRPS who also demonstrated correlations between PINP levels and scintigraphy ratios. (A) Pairwise comparisons between AP levels in healthy controls (HC) and subgroups of patients with cold and warm CRPS. Lines indicate values as medians and the first and third quartile. Differences between groups were calculated using the Kruskal–Wallis test and Dunn post-hoc test with Bonferroni correction. (B and C) Correlations between scintigraphy ratios and MCP and PIP regions in the warm CRPS subtype. Calculations with Spearman correlations were adjusted for multiple testing using Bonferroni correction. PINP, procollagen type 1 N-terminal propeptide; metacarpophalangeal and prox. interphalangeal subregions of the hands, MCP, PIP.

All examined bone serum markers are depicted in a schematic illustration (Fig. 3A). The median serum AP levels were significantly higher in patients (Fig. 3B), and this difference persisted after matching the groups (Supplementary Table S2, http://links.lww.com/PR9/A267). Among the CRPS temperature subtypes, only patients with warm CRPS exhibited elevated AP values compared to those with cold CRPS or HC (Fig. 4A). However, AP was not correlated with other specific bone serum markers or TPBS ratios (all *P*-values >0.2). Higher 25-OH vitamin D levels were initially observed in patients than in HC (Fig. 3C), but this difference was not significant after adjusting for age and sex (Supplementary Table S2, http://links.lww.com/PR9/A267). The serum levels of the other specific bone markers, OPG, PINP, and β-CTx, did not differ significantly between patients and HC or among the temperature subtypes (Figs. 3D–F; Table 2).

Most bone serum markers did not correlate with scintigraphy ratios in the 41 patients who underwent TPBS. Specifically, the correlations between PINP and scintigraphy ratios at the MCP and PIP sites resulted in Spearman ρ = 0.40, *P* = 0.069 and Spearman ρ = 0.381, *P* = 0.092, respectively. However, stratification by temperature subtype revealed a significant positive correlation between the MCP ratio and PINP in the warm subtype (Fig. 4B), although it was not significant for the PIP ratio and PINP (Fig. 4C). After adjusting for age and sex, significant correlations were found only within the female subgroup between the MCP ratio and serum PINP levels (Supplementary Table S3, http://links.lww.com/PR9/A267). These results emphasise the specific context in which TPBS and bone serum markers are correlated, indicating that the underlying mechanisms of CRPS may vary substantially among subtypes and individuals.

In addition, an exploratory analysis investigating the relationship between immobilisation duration and bone serum markers revealed no correlations (Supplementary Fig. S2A-E, http://links.lww.com/PR9/A267), and scatterplots comparing storage duration (ie, time delay between blood sampling and analysis) to serum values showed no discernible trends.

## 4. Discussion

This study assessed the prevalence of disturbed bone metabolism in patients with CRPS using a novel image segmentation technique for TPBS. Automated analysis revealed widespread radiotracer enhancement across CRPS subtypes, indicating that altered bone metabolism is a common feature of CRPS, even when it is not visually detectable. This finding emphasises the potential of automated image analysis as a valuable diagnostic tool. Although most bone serum markers showed no significant differences and may not replace TPBS or serve as standalone biomarkers, they still offer valuable insights when used in conjunction with imaging techniques. This is highlighted in the case of warm CRPS among female patients, where a positive correlation between MCP scintigraphy ratios and serum PINP levels indicates enhanced osteoblast activity. Furthermore, elevated AP levels in warm CRPS that did not correlate with other bone serum markers or TPBS ratios, indicate distinct pathophysiological processes beyond bone metabolism disturbances in this subtype.

### 4.1. Automated segmentation and quantification of three-phase bone scintigraphy

Novel automated segmentation detected radiotracer accumulation in nearly all the examined patients. The absence of a control group of patients with distal fractures but no CRPS limited our ability to precisely determine the CRPS-specific radiotracer enhancement. A previous study using manual annotation and quantification of the MCP region in patients with CRPS, compared to a fracture control group, reported a scintigraphy ratio cut-off of 1.32, with 69% sensitivity and 75% specificity.^49^ Applying this cut-off to our cohort identified 16 of 28 patients (57%) as positive for CRPS, indicating specific alterations in the majority. Increased bone turnover may clinically present as a reduced threshold for deep pressure pain, with the measurement location being crucial. In this study, PPT measurements above the thenar muscle did not correlate with TPBS ratios, consistent with previous findings where only deep pain thresholds measured directly over joints, such as the MCP, correlated with radiotracer enhancement.^26^

The underlying cause of increased bone turnover in patients with CRPS is not yet fully understood. The absence of higher accumulation ratios in 9 patients (32%) with direct hand injuries and the lack of correlation between immobilisation duration and radiotracer enhancement suggest that distal bone metabolism alterations may develop independently of direct locoregional bone damage. This implies the involvement of additional factors, such as neurogenic inflammation, particularly in the early stages of the disease. Neuropeptides like substance P and CGRP, released by sensitised nociceptive C-fibres,^24^ lead to neuroinflammation with increased cytokine production (TNF-α, interleukin-6) and proliferation or immigration of Langerhans and mast cells.^4,15^ The presence of IgG antibodies against autonomic nervous system receptors (β2-, α1A-adrenergic, and M2-muscarinic receptors) suggests an autoimmune component in some patients with CRPS.^10,20^ These mechanisms may collectively contribute to varying degrees of bone loss, independent of injury location and CRPS type. Neuropeptides can stimulate receptor activator of nuclear factor kappa-Β ligand (RANKL) production, leading to osteoporotic processes,^50^ while TNF-α further enhances osteoclast formation and bone resorption.^27^ An in vitro study showed that β2-adrenoreceptor activation promotes osteoclast formation,^1^ and an osteoarthritis animal model demonstrated that osteoclast activation leads to netrin-1–mediated bone remodelling and sensory pain–provoking innervation,^51^ potentially reflected in the lower deep pain threshold in the affected joints in humans.^26^ Ultimately, inflammatory and autoimmune processes that activate osteoclasts and osteoblasts are likely to contribute to the deep pressure pain observed in many patients with CRPS.

### 4.2. Bone serum markers

This study found few differences between patients with CRPS and HC regarding the serum markers OPG, PINP, and β-CTx, challenging their potential to replace TPBS or serve as standalone biomarkers. Our inability to replicate earlier differences in OPG levels may be attributed to our larger and more diverse cohort, fewer patients with fractures, or variations in the enzyme-linked immunosorbent assays used. Other factors, such as diurnal fluctuations affecting β-CTx, which are influenced by food intake,^5^ were controlled by collecting all blood samples in the morning after overnight fasting. However, serum markers reflect general bone turnover, varying widely among individuals based on age, sex, hormone profile, diet, and lifestyle, leading to high interindividual variability, as evidenced by broad reference values for PINP and β-CTx.^28,46^ Localised changes may not necessarily lead to systemic biomarker elevation, as observed in rheumatoid arthritis.^43,48^ Regardless, 25-OH vitamin D levels were not consistently decreased in the CRPS cohort compared to healthy controls. While vitamin D deficiency remains a concern, vitamin D supplementation should be tailored to specific needs rather than routinely applied in patients with CRPS.

### 4.3. Warm and cold complex regional pain syndrome subtypes

Higher serum AP levels were observed in the warm CRPS subtype without correlation with TPBS ratios or other bone serum markers, suggesting broader pathophysiological processes. Alkaline phosphatase is known for its role in bone health, but it is also implicated in various inflammatory conditions such as atherosclerosis.^11^ Recent studies have highlighted the involvement of AP in anti-inflammatory processes through its ectonucleotidase activity, which converts proinflammatory ATP into the anti-inflammatory nucleotide adenosine.^17,31,32^ Thus, elevated AP levels in warm CRPS may reflect a combination of increased bone remodelling and (anti-)inflammatory processes characteristic of this subtype.

Furthermore, while most bone serum markers did not correlate with TPBS, a positive correlation between MCP TPBS ratios and serum PINP levels was observed in female patients with warm CRPS, indicating increased osteoblast activity detectable by both measurements. In the future, PINP could be examined alongside TPBS ratios to assess and monitor bone metabolism, similar to osteoporosis management. Procollagen type I N-terminal propeptide is especially interesting in this regard given its minimal circadian fluctuations, thus eliminating the need for morning and fasting state sampling.^29,43^ Regarding TPBS ratios, our newly developed software enables simple radiotracer quantification, facilitating future research in a user-friendly manner.^37,38^

### 4.4. Limitations and strengths of the study

The sample size of 41 patients undergoing TPBS, introduced after the study began, may limit the generalisability of the results. Furthermore, the absence of a control group limits the direct comparative analysis of patients with CRPS to those with normal healing after trauma,^8^ crucial for validating CRPS-specific changes. Heterogeneity in image acquisition prevented the establishment of standardised subregions in the feet, possibly affecting the measurement precision. The partial overlap of warm CRPS with an early inflammatory subtype may complicate subtype differentiation.^18^ This was somewhat mitigated by similar times since the triggering event in cold and warm subtypes. While a commonly used 1°C cut-off was applied to stratify temperature groups, more rigorous criteria are required in the future to clearly distinguish temperature subtypes and enhance diagnostic accuracy. Despite these limitations, the robust sample size for bone serum markers and meticulously standardised prospective data collection provide considerable reliability and validity for our results. Our novel methodology for assessing bone metabolism in CRPS shows promise but requires further validation before clinical application.

## 5. Conclusions

Using an automated image segmentation technique for TPBS image analysis, this study revealed widespread radiotracer enhancement in the affected extremities, suggesting frequent alterations in bone metabolism in CRPS. These disturbances can be quantified automatically in a standardised manner beyond conventional visual inspection. As alterations were evident across CRPS subtypes and injury locations, patients with CRPS may exhibit altered bone metabolism irrespective of these factors. Unique patterns in the warm CRPS subtype, marked by higher serum AP levels and a correlation between MCP radiotracer enhancement and serum PINP in female patients, indicate distinct pathophysiological mechanisms. These observations highlight the potential of integrating advanced image analysis with serum marker assessment to enhance the diagnostic precision and patient stratification for the development of targeted therapies.

## Disclosures

The University Hospital Wurzburg and HR received patient fees from Gruenenthal and Algiax for the clinical trials. The authors declare that they have no conflicts of interest.

## Appendix A. Supplemental digital content

Supplemental digital content associated with this article can be found online at http://links.lww.com/PR9/A267.
