# Supplementary material for: Bone metabolism in complex regional pain syndrome
Source: Pain Rep. 2024 Nov 20;9(6):e1217. doi: 10.1097/PR9.0000000000001217 (PMC11581760; doi:10.1097/PR9.0000000000001217)
Supplement: Supplementary file 1 [file painreports-9-e1217-s001.pdf]

Supplementary materials

Figure S1

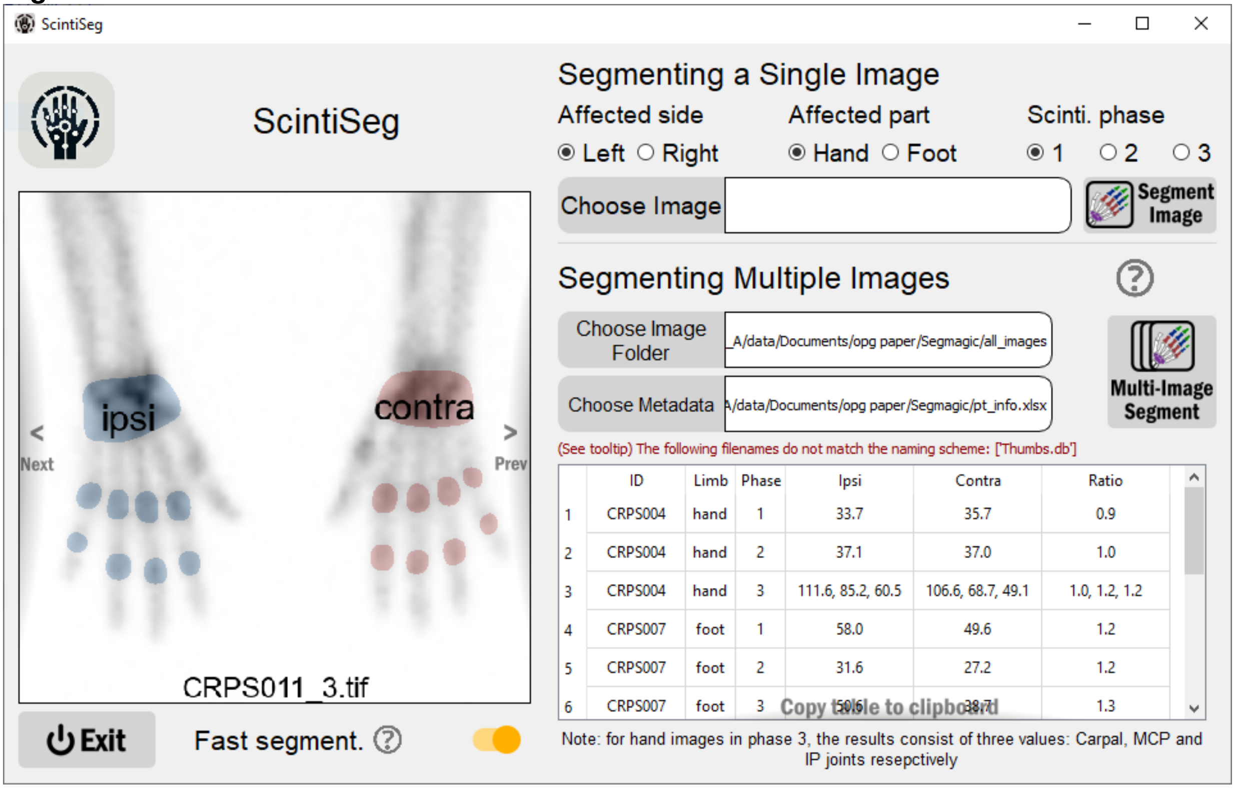

Figure S1: GUI of the newly developed software for automated quantification of TPBS images. For single-image analysis, they can be loaded per drag-and-drop into the window on the left and segmented after indication of relevant information (side, extremity, and phase). An entire folder with images can also be used for multiple analyses and the output can be copied and pasted onto a spreadsheet or database.

Figure S2

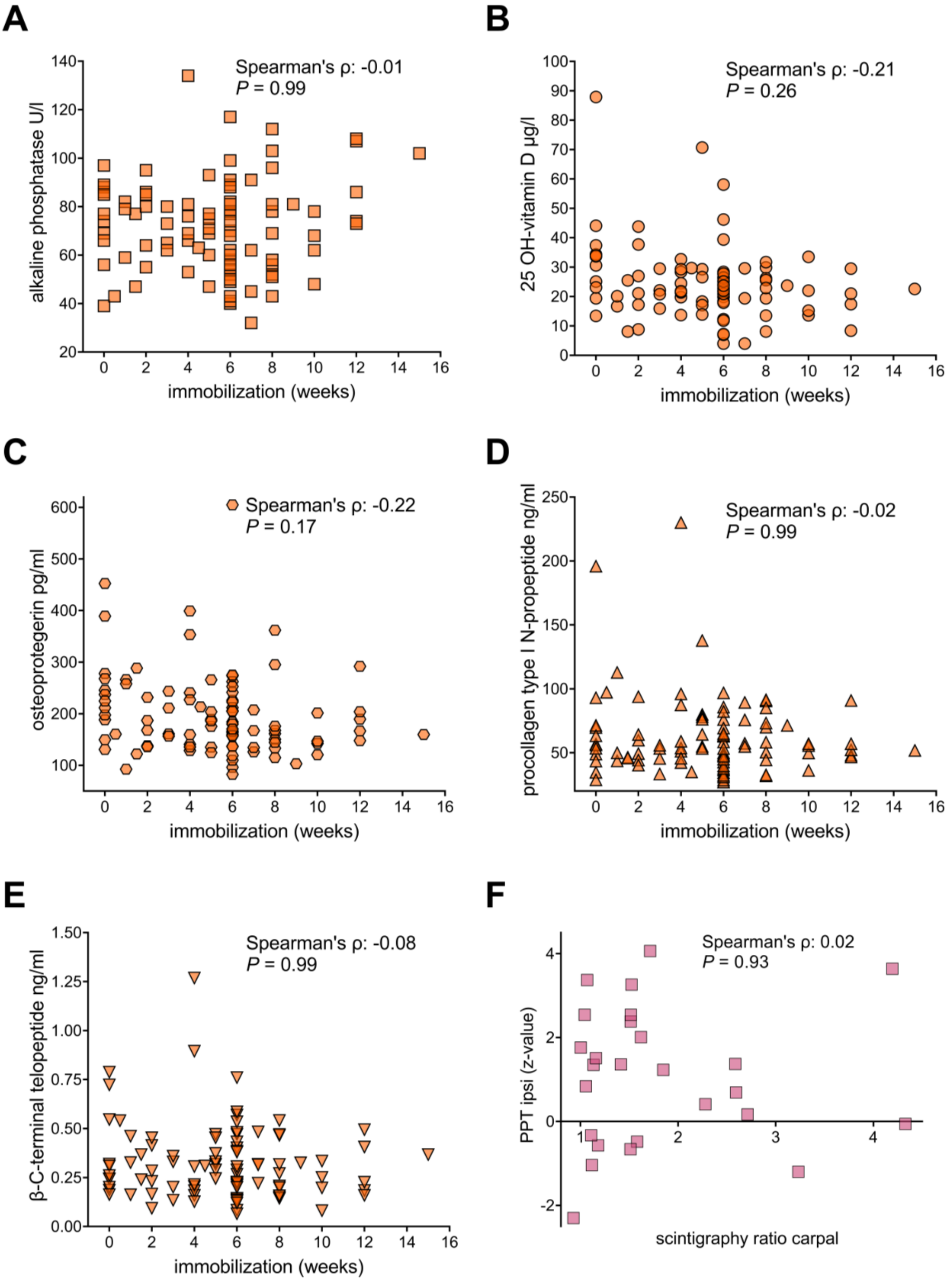

Figure S2: Immobilisation does not correlate with bone serum marker levels. (A-E) Scatter plots displaying immobilisation duration and bone serum markers. Calculations with Spearman correlations were adjusted for multiple testing using Bonferroni correction. (F) Scatter plot showing z-transformed ipsilateral Pressure Pain Threshold (PPT) and scintigraphy ratios for the carpal region.

Figure S3

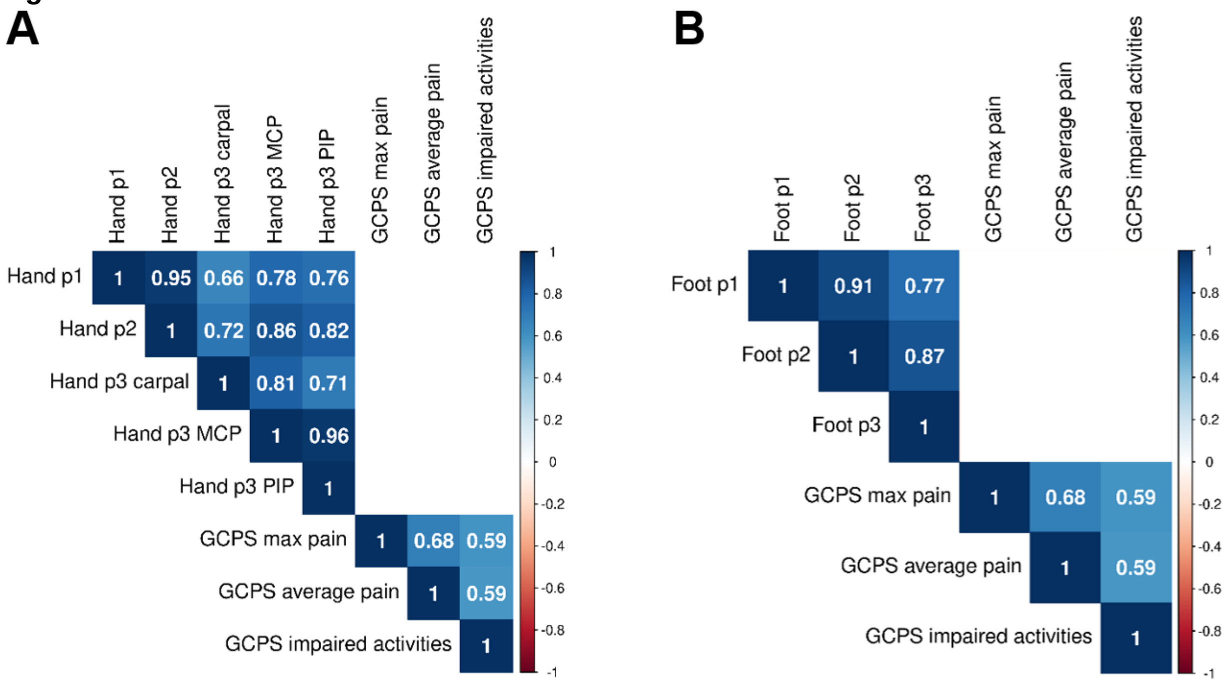

Figure S3: Radiotracer accumulation does not correlate with pain perception. Spearman correlation plots illustrating the relationships between scintigraphy ratios and pain parameters. (A) Hand regions with phases 1-3 of scintigraphy: p1-3; carpal, metacarpophalangeal (MCP), and proximal interphalangeal (PIP) subregions of the hands. (B) Foot regions with phases 1-3 of scintigraphy: p1-3. Correlation coefficients (ρ) are displayed inside the intersections (white), with only significant correlations shown ($P < 0.05$).

Table S1

Model	Test image	Uncertainty score	Dice score
whole hand p1-2	hand p1-2 #1	0.142243	0.944982
	hand p1-2 #2	0.094711	0.967074
	hand p1-2 #3	0.128376	0.933574
subregions hand p3	hand p3 carpal #1	0.218703	0.866673
	hand p3 MCP #1	0.336767	0.853725
	hand p3 PIP # 1	0.419903	0.764353
	hand p3 carpal #2	0.246439	0.887604
	hand p3 MCP #2	0.337525	0.87317
	hand p3 PIP #2	0.417616	0.787467
whole foot p1-3	foot #1	0.101376	0.958546
	foot #2	0.067058	0.967071
	foot #3	0.144839	0.936965
	foot #4	0.070694	0.949326

Table S1: Good performance in uncertainty scores and congruence of final models on test images. Phases 1-3 of scintigraphy, p1-3; carpal, metacarpophalangeal, and prox. interphalangeal subregions of the hands, carpal, MCP and PIP.

Table S2

	Fracture	Surgery	minor trauma	other	*P*-value	q-value
Hand p1	1.16 (1.01, 1.47)	1.34 (1.04, 1.51)	1.03 (0.95, 1.16)	1.17 (1.11, 1.23)	0.58	0.58
Hand p2	1.28 (1.04, 1.47)	1.29 (1.03, 1.59)	1.05 (1.01, 1.07)	1.19 (1.12, 1.25)	0.52	0.58
Hand p3 carpal	1.60 (1.51, 2.59)	1.77 (1.18, 2.28)	1.06 (0.93, 1.12)	1.08 (1.04, 1.11)	0.022	0.17
Hand p3 MCP	1.69 (1.14, 2.12)	1.91 (1.13, 2.35)	1.06 (1.02, 1.22)	1.39 (1.22, 1.56)	0.28	0.58
Hand p3 PIP	1.61 (1.07, 2.00)	1.97 (1.10, 2.18)	1.08 (0.97, 1.29)	1.52 (1.22, 1.82)	0.42	0.58
Foot p1	1.27 (1.06, 1.43)	0.95 (0.87, 1.16)	NA	1.21 (0.95, 1.48)	0.40	0.58
Foot p2	1.27 (0.91, 1.43)	0.94 (0.86, 1.16)	NA	1.20 (0.99, 1.40)	0.47	0.58
Foot p3	1.59 (1.24, 1.82)	1.28 (0.98, 1.29)	NA	1.63 (1.53, 1.73)	0.22	0.58

Table S2: No difference in radiotracer accumulation stratified by injury. Data are displayed for upper (n = 28) and lower extremity (n = 13) and were analysed using the Kruskal-Wallis test with post-hoc False Discovery Rate. Phases 1-3 of scintigraphy, p1-3; carpal, metacarpophalangeal, and prox. interphalangeal subregions of the hands, carpal, MCP and PIP.

Table S3

	**CRPS**<br>N = 48	**HC**<br>N = 48	***P*-value**
Age	52 (38, 60)	52 (37, 60)	0.8
Female	26 (54%)	26 (54%)	>0.9
Alkaline phosphatase U/L	70 (56, 83)	60 (53, 69)	0.039
25-OH vitamin D µg/l	21 (14, 29)	17 (11, 21)	0.059
OPG pg/ml	182 (148, 227)	194 (146, 240)	0.9
PINP ng/ml	58 (49, 77)	50 (44, 65)	0.081
β-CTx ng/ml	0.31 (0.20, 0.45)	0.34 (0.21, 0.43)	0.6

Table S3: Alkaline phosphatase retained differences between age- and sex-matched groups. Values are medians (1st, 3rd quartile) and absolute and relative frequencies n (%). Data were analysed using the Mann–Whitney U test or Pearson's chi-squared test. CRPS, Complex Regional Pain Syndrome; CSS, CRPS Severity Score; AP, alkaline phosphatase; OPG, Osteoprotegerin; PINP, procollagen type I N-propeptide; β-CTx, β-C-terminal telopeptide.

Table S4

	**ρ for MCP**	***P*-value**	**ρ for PIP**	***P*-value**
No control	0.60	0.02	0.54	0.04
Age	0.65	0.02	0.60	0.03
Sex (Male)	0.40	0.6	0.40	0.6
Sex (Female)	0.72	0.01	0.65	0.03

Table S4: Female sex was the most important factor contributing to the correlation between serum PINP and scintigraphy. Correlations between PINP values and MCP and IP signals in warm CRPS after controlling for the effects of age and sex. Scintigraphy for phase 3 of the metacarpophalangeal and prox. interphalangeal region, MCP, PIP; procollagen type I N-propeptide, PINP. Spearman correlations. After the Bonferroni correction, the α-level was set at .025.
